# Pediatric Cancer Outcomes in an Intensive Care Unit in Pakistan

**DOI:** 10.1200/JGO.18.00215

**Published:** 2019-03-12

**Authors:** Gull Zareen Khan Sial, Saadiya Javed Khan

**Affiliations:** ^1^ Shaukat Khanum Memorial Cancer Hospital and Research Center, Lahore, Pakistan

## Abstract

**PURPOSE:**

Although cancer is uncommon, it is a significant cause of pediatric morbidity and mortality in the developing world. The need for intensive care in pediatric oncology has increased with more intense chemotherapeutic interventions. It is important to identify patients who will benefit from management in the intensive care unit (ICU), given the resource limitation in developing countries. In this review, we examine our institutional experience with pediatric patients with cancer needing ICU care.

**METHODS:**

A retrospective chart review from December 2015 to June 2017 was performed with institutional review board approval for all pediatric oncology patients admitted to the ICU. Data collection included age, diagnosis, disease stage, Pediatric Risk of Mortality (PRISM III) score, and therapeutic interventions.

**RESULTS:**

We reviewed 59 pediatric oncology ICU medical records. There were 36 boys (61%) and 23 girls (39%). The median age was 4 years. Average stay in the ICU was 4.6 days. Three significant reasons for ICU referral were respiratory distress, sepsis, and circulatory collapse. There were 34 ICU survivors (57.6%). Among those who survived the ICU, 20 patients (58.8%) later died of therapy-related complications. Factors related to increased ICU mortality included the need for mechanical ventilation, the need for inotropic support, the number of failing organs, and a high PRISM III score.

**CONCLUSION:**

The mortality rate for pediatric oncology patients admitted to the ICU in developing countries is higher than in developed countries. Mortality was significantly related to the need for mechanical ventilation. PRISM III scoring can help identify patients who can benefit from ICU treatment, which is expensive in resource-limited low- and middle-income countries such as Pakistan.

## INTRODUCTION

Cancer in children, although rare, is the leading cause of death after the age of 1 year in the United States.^1^ The overall survival for children with cancer has improved over the last five decades. In 1975, slightly more than 50% of children diagnosed with cancer younger than 20 years of age lived for at least 5 years.^2^ Data from 2007 to 2013 show that 83% of children younger than 20 years of age with cancer lived for at least 5 years.

This advancement in survival can be explained by the development of new curative drugs during the 1960s to 1970s, such as vincristine, cytarabine, doxorubicin, and asparaginase.^3^ It can also be explained by improvement in supportive care, such as identifying the need to treat children with fever or neutropenia,^4^instituting preventive measures for opportunistic organisms,^5^ and administering blood products in case of myelosuppression.^6^

Current chemotherapy protocols subject to risk stratification are more successful but can cause considerable therapy-related morbidity and mortality.^7,8^ Some of the complications can be managed on the inpatient wards, but problems such as tumor lysis syndrome, respiratory failure, circulatory collapse, and so on, require admission to an intensive care unit (ICU).^9^ Approximately one of every three to four children diagnosed with cancer are admitted to the pediatric ICU (PICU) at least once during their illness. There are reports of poor outcomes in children with malignancies requiring mechanical and/or inotropic support.^10-12^

Although cancer is uncommon, it is a significant cause of pediatric morbidity and mortality in developing countries. Survival approximations vary between 10% and 50%.^13^ A major limiting factor in survival improvement is the cost and availability of specialized services in low and middle-income countries (LMICs) such as Pakistan. It is essential to treat appropriately and identify patients who will benefit from ICU care. Intensive care can be expensive in resource-limited settings. The Pediatric Risk of Mortality (PRISM) score and its updated version, PRISM III, includes physiologic as well as laboratory variables to help predict morbidity and mortality risk. In this analysis, we assessed the causes leading to intensive care transfer, interventions, and survival in the ICU. In addition, we evaluated the PRISM III score as a tool to predict ICU survival.

## METHODS

This retrospective study was performed at Shaukat Khanum Memorial Cancer Hospital in Lahore, Pakistan. This is the only freestanding cancer hospital in the country. Pediatric oncology is one of the subspecialties caring for approximately 400 new patients per year. There are 34 inpatient beds for children. The 10-bed ICU in the hospital does not have the advantage of a pediatric intensivist. Pediatric patients are cared for by physicians who normally treat adults.

We reviewed the charts of 59 patients admitted to the ICU from December 2015 to June 2017 after approval from the hospital institutional review board. The department treats children 18 years of age or older. Data collected included age, sex, weight, primary malignancy, remission status, reason for admission to the ICU, PRISM III score, neutropenia, number of organ failures, therapeutic interventions needed, length of ICU stay, outcome at the time of leaving the ICU (ICU survivor *v* ICU nonsurvivor), and the overall outcome of the patient at the last follow-up.

Organ system dysfunction and sepsis were defined according to the international pediatric sepsis consensus conference: definitions for sepsis and organ dysfunction in pediatrics.^14^ Severity of illness was assessed by the PRISM III score obtained on the day of admission to the ICU.^15^ Bacterial and viral infections were confirmed on blood culture and polymerase chain reaction results. Invasive fungal infections were diagnosed on computed tomography with *Aspergillus* galactomannan and/or beta-d-glucan detection assays.

Neutropenia was defined as an absolute neutrophil count of less than 500 cells per μL. Mechanical ventilation was started in cases of tachypnea, cyanosis, or respiratory failure. Inotropic support was defined as vasopressors that were used for circulatory collapse within 24 hours of ICU admission. Patients were classified as ICU survivors if they were alive at the time of discharge from the ICU versus ICU nonsurvivors. Overall mortality was estimated depending on the patient’s outcome at the last follow-up. Statistical analysis was performed using the SPSS 10.0 statistical software (SPSS, Chicago, IL). Fisher’s exact test was used to test ICU survivorship with different risk factors.

## RESULTS

During the study period, 611 new pediatric oncology patients were seen at the hospital. Among these were 247 patients with lymphomas, 215 with solid tumors, and 149 with leukemia. Average age at diagnosis of cancer was 6 years. The total number of children admitted to the ICU during this period was 59 (9.6%).

There were 36 males (61%) and 23 females (39%). The median age was 4 years, with an interquartile range value of 3 years (range, 1 to 17 years). The median length of stay in the ICU was 3 days, with an interquartile value of 4 days (range, 1 to 26 days). A total of 37 children (62.7%) were diagnosed with hematologic malignancies, and 22 (37.3%) were diagnosed with noncentral nervous system solid tumors. CNS solid tumors are not treated at our institution.

There is not much data available about the impact of malnutrition on ICU mortality. We feel that this is a relevant parameter in LMIC settings such as ours. We used weight for age to assess its effect on ICU mortality. As listed in Table 1, more children (47.8%) died in the ICU whose weight for age was less than the third percentile compared with those at the third percentile or above (38.9%).

**TABLE 1:**
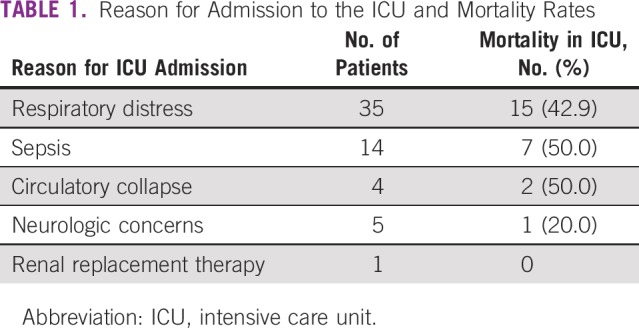
Reason for Admission to the ICU and Mortality Rates

The three main reasons for transferring patients to the ICU were respiratory distress (59.3%), sepsis (23.7%), and circulatory collapse (6.8%), as listed in Table 2. The highest mortality was seen in children who were admitted with sepsis (50%) or circulatory collapse (50%), followed by respiratory distress (42.9%). Four patients with respiratory distress had proven fungal infections (11.4%), whereas five (35.7%) patients with sepsis had proven microbiologic etiologies, including, *Pseudomonas*, candidemia, methicillin-resistant *Staphylococcus aureus*, *Stenotrophomonas*, and *Enterobacter* blood infections.

**TABLE 2:**
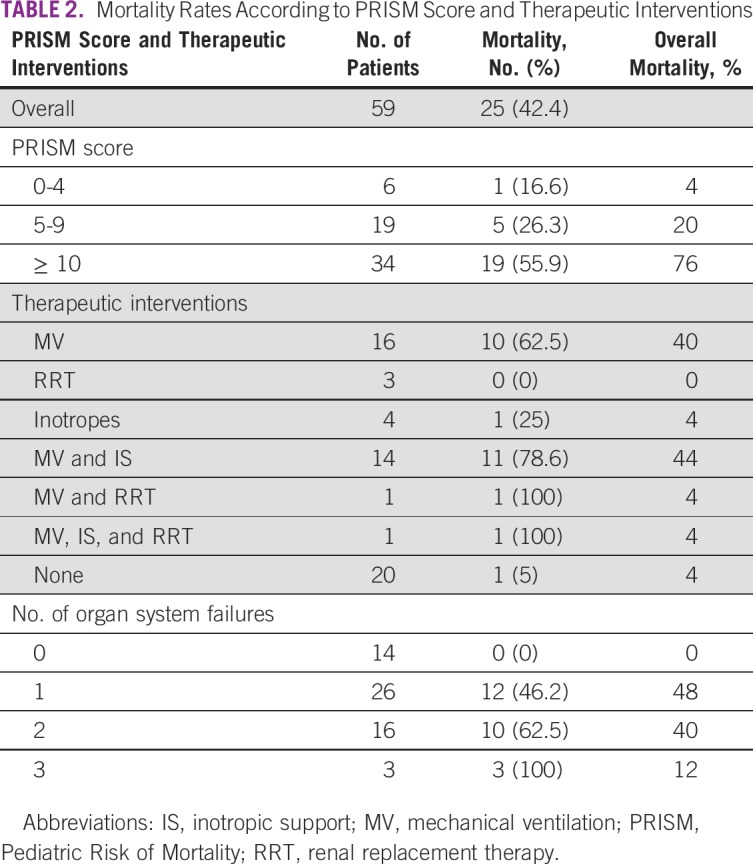
Mortality Rates According to PRISM Score and Therapeutic Interventions

Average stay in the ICU was 4.6 days. During the ICU stay, 32 patients (54.2%) were ventilated mechanically. Sixteen of these also needed inotropic support. One patient needed both mechanical ventilation and renal replacement therapy, whereas one of these needed all three interventions (Table 3). Both patients requiring either all three interventions (n = 1) or mechanical ventilation and renal replacement therapy (n = 1) died. Eleven children (78.6%) requiring both mechanical ventilation and inotropic support died. Survival of children without any organ system compromise was excellent but worsened with increasing organ system failure.

**TABLE 3:**
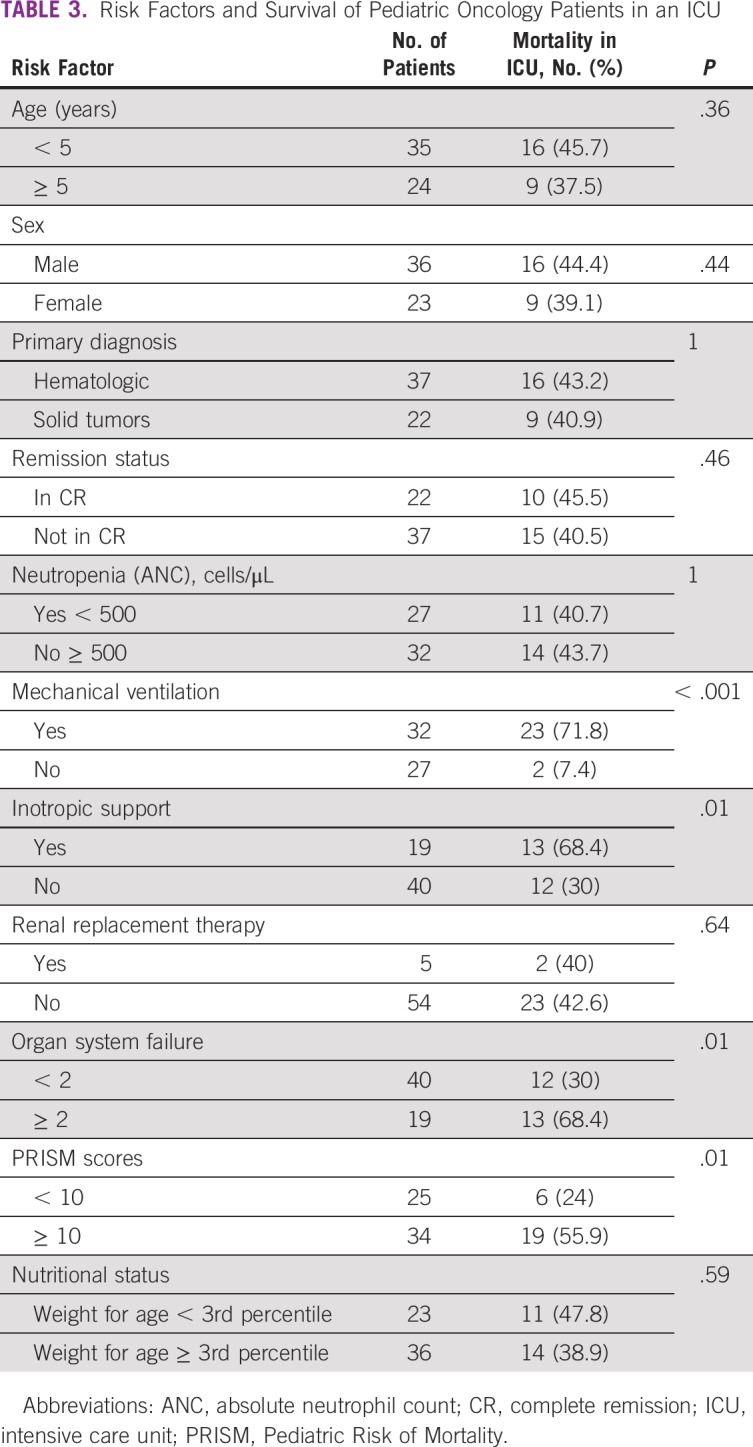
Risk Factors and Survival of Pediatric Oncology Patients in an ICU

Significant associations with ICU mortality are listed in Table 3. There was no significant difference seen in age, sex, primary diagnosis, remission status, neutropenia, and renal replacement therapy when ICU survivors were compared with ICU nonsurvivors. Of the 59 pediatric oncology patients transferred to a higher level of care, 25 (42.4%) could not survive in the ICU. The mortality rate was significantly related to mechanical ventilation (*P* < .001), inotropic support (*P* = .01), and two or more organ system failures (*P* = .01).

On admission to the ICU, the mean PRISM III score was 11.9 ± 7.9. The mean PRISM III score among survivors was significantly lower than that among nonsurvivors (8.9 ± 4.3 *v* 16 ± 9.5; Student *t* test, t = −3.8; *P* < .001). The mortality rate was 24% for those with a PRISM III score of less than 10 and 76% for patients with scores of 10 or greater. For this review, we considered a PRISM III score of 10 or greater as high.^16^

## DISCUSSION

This retrospective review helped us evaluate factors that led to higher mortality rates in pediatric oncology patients admitted to the ICU. From December 2015 to June 2017, 611 children were diagnosed with cancer at our institution. Of these, 59 (9.6%) needed a higher level of care in the ICU. Because of a scarcity of subspecialty-trained pediatric intensivists, we do not have a dedicated PICU at the hospital. Physicians who normally treat adults are treating pediatric oncology patients. We cannot be sure if some of the ICU mortality could have been attributed to difficulties in securing beds expeditiously on the adult ICU, given that there are no dedicated pediatric beds in the unit.

In this review, we excluded admissions whose acuity of illness was relatively low, such as postsurgical or postprocedural observations. The majority of the patients referred to us are malnourished and come from far-flung areas with a delayed diagnosis. Our mortality rate of 42.4% is close to figures reported in older literature^17-19^ but definitely higher than in newer studies.^20^ Published statistics are from the PICU, whereas our report is from an adult ICU, given the limitations in our country. Considering that we treat all diagnoses except for brain tumors, most patient issues are addressed in the regular pediatric oncology unit.

In a report on 32 patients in a PICU in Homburg, Germany, Meyer et al^21^ showed an overall survival of approximately 78%. Their mortality rate was significantly related to hematologic malignancy, neutropenia, mechanical ventilation, inotropic support, and number of organ failures. Our study with a similar number of patients showed similar results, except that hematologic malignancies and neutropenia were not seen to be a risk factor for poor survival. We believe this is because our subsets of hematologic malignancies are mostly standard risk. Most high-risk patients coming to our hospital are sick with organ compromise by the time they reach us and die despite our best efforts at resuscitation. In our institutional experience, the tendency to abandon treatment is higher in families taking care of children with high-risk lymphomas, solid tumors, and leukemia. The Indus hospital in Karachi recently published data indicating higher abandonment rates in solid tumors as well.^22^ Otherwise, we have an extremely low threshold for admission and administering antimicrobials when these patients present with fever in the setting of neutropenia with an absolute neutrophil count of less than 500 cells per μL or with a decreasing trend.

In our cohort, four factors were identified to be significantly associated with poor survival: mechanical ventilation, inotropic support, number of organ failures, and high PRISM III score.^11,22^ Mechanical ventilation and inotropic support combined resulted in a high mortality rate (78.6%). Hallahan et al^23^ studied 206 admissions to the PICU in a 9-year period and reported a 54% survival rate in patients needing both. Our poor results can be explained by the inadequacy of facilities in our part of the world. We do not have access to noninvasive ventilation, such as continuous positive airway pressure equipment that can be used on the pediatric floor in an attempt to prevent ICU transfer for invasive ventilation. Nor do we have oscillators and chest physiotherapy vests for better respiratory hygiene.^24^ Conventional ventilators were used under the supervision of adult intensivists.

The limitation with this study is the retrospective nature of analysis and the small number of patients. Adult ICU physicians with no history of pediatric training care for these sick children. In developing countries with infrastructure limitations such as ours, children with cancer do not have easy access to a pediatric intensivist. Another important issue is finances. The per capita income in US dollars was $1,629 in fiscal year 2017, according to the Pakistan Economic Survey report generated for 2016 to 2017. This figure is meager compared with the cost of an average day stay in the ICU at our hospital (approximately $400) or at other private settings within Pakistan.

Wherever possible, we have adapted international chemotherapy protocols to help reduce the intensity and minimize toxicity without compromising too much on the outcomes of the disease. The department has also attempted to anticipate and institute management measures to reduce ICU transfers. A prime example is our use of rasburicase to prevent tumor lysis syndrome in patients with lymphoma/leukemia with high disease burden. But here again, the high cost of rasburicase is a concern; hence, we only have the budget to treat 10 patients per year. We have an institutional pediatric algorithm whereby rasburicase is used to prevent patient transfer to the ICU for dialysis.

PRISM III score was significantly associated with ICU mortality. A large study from the ICU at St. Jude Children’s Research Hospital suggested that the PRISM III scoring system is an appropriate tool for predicting patient mortality risk.^25^ In our setting, PRISM III scores helped to identify high-risk patients. The need for both ventilation and inotropic support are key aspects in assessing the risk of mortality. We want to prospectively study PRISM III score application for more precise prognostication in resource-limited settings such as ours. ICU care is expensive and in LMICs, prudent goals of treatment at the start of ICU admission are essential. We do not want to overlook patients who will benefit from ICU care but, at the same time, patients who are sick with high PRISM III scores need to have an efficient care plan where cost versus benefit from ICU care is assessed judiciously.

Our overall outcomes are not encouraging despite successful ICU outcomes. This can be due to multiple factors, including the poor nutritional status of children with cachectic cancer conditions in LMICs. Rehabilitation with total parenteral nutrition is expensive and not always available. We improve nutrition using nasogastric tubes, which takes time. Our association of malnutrition with ICU mortality was not statistically significant, possibly because of the small number of patients in the analysis. We believe that achieving adequate growth parameters is crucial to better tolerance of chemotherapy and its adverse effects, and that this needs to be studied in a prospective manner.^26^

Among other factors leading to higher overall mortality at our institution are delays in diagnosis and poor infrastructure to support timely transfer of care. Most of our patients are coming from faraway northern areas of the country as well as from Afghanistan. We are making all possible efforts to create awareness about the importance of early diagnosis with primary care physicians and stress the importance of multidisciplinary treatment of pediatric cancer in Pakistan, both at an institutional and national level.
